# Social Isolation And Frailty Among Chinese Older Adults: Role Of Dietary Patterns

**DOI:** 10.1093/geroni/igaf122.3845

**Published:** 2025-12-31

**Authors:** Zhongfei Pei, Ming Xu

**Affiliations:** Peking University, Beijing, Beijing, China (People’s Republic); Peking University School of Public Health, Beijing, Beijing, China (People’s Republic)

## Abstract

**Background:**

Frailty is a health problem that is extremely prone to arise with aging and is more prevalent in socially isolated older adults. The dietary pattern often serves as an important determinant of health in later life that cannot be ignored. Identifying the role that a specific dietary pattern plays in social isolation and frailty can provide insights to improve the quality of life of older adults.

**Methods:**

This cross-sectional study used data from the eighth wave of the Chinese Longitudinal Healthy Longevity Survey (CLHLS) conducted in 2018. A total of 8889 older adults (aged 60+) were included in our study. Frailty was assessed using FRAIL Scale. Social isolation was defined by marital status, living arrangement, frequent visit by family members, and social participation. Dietary patterns were explored by principal component analysis. The logistic regression was used to carry out the moderating effect analysis.

**Results:**

Four dietary patterns were identified, namely “vegetable-nut-mushroom or alga”, “egg-milk”, “meat-fish”, and “pickle”. Subjects with higher levels of social isolation were associated with a higher risk of frailty (OR = 2.31, 95%CI=2.17-2.46). We observed that the egg-milk pattern as a moderator alleviated this relationship (OR = 0.91, 95%CI=0.86-0.97).

**Conclusions:**

Our study suggested that social isolation was positively correlated with frailty and the egg-milk pattern is a moderator in this relationship. Improving the social isolation of older people was beneficial to prevent frailty. It is necessary to advocate the dietary pattern characterized by eggs and milk in socially isolated older adults to reduce the risk of frailty.

